# Supplementary material for: Span-based single-stage joint entity-relation extraction model
Source: PLoS One. 2023 Feb 7;18(2):e0281055. doi: 10.1371/journal.pone.0281055 (PMC9904475; doi:10.1371/journal.pone.0281055)
Supplement: Attachment — Submitted filename: Response to Reviewers.pdf [file pone.0281055.s002.pdf]

Editor-in-Chief of *PLOS ONE*

Dear editor and reviewers

On behalf of all the contributing authors, I would like to express our sincere appreciations of your letter and reviewers' constructive comments concerning our article entitled "Span-Based Single-Stage Joint Entity-Relation Extraction Model" (Manuscript No.: PONE-D-22-22843R1). These comments are all valuable and helpful for improving our article. According to the associate editor and reviewers' comments, we have made extensive modifications to our manuscript and supplemented extra data to make our results convincing. In this revised version, changes to our "Revised Manuscript with Track Changes" were all highlighted within the document by using yellow-colored text. Point-by-point responses to the nice associate editor and two nice reviewers are listed below this letter.

To Editor:

1. We try to make our manuscripts conform to the style requirements of PLOS ONE, at the same time, the Latex source file containing the .tex file will be uploaded together.
2. The code of this project has been opened to GitHub, and the address is also mentioned in the paper.
3. "Funding Information" has been modified.
4. Author information has been modified.

To Reviewer #1:

1. The limitations and shortcomings of the method are written in lines 328 "Although ...".
2. Follow your advice and add a contribution note at the end of the introduction.
3. A detailed comparison with other methods is made in the "Validation Experiences" section.
4. Follow your suggestions and add a discussion on future work in the conclusion section.

To Reviewer #2:

1. This method is not only for Chinese. We supplement the experiment and results on NYT dataset in our paper.
2. Follow your suggestions and the code of this project has been opened to GitHub, and the address is also mentioned in the paper.
3. Following your suggestions, I changed the description of the super parameters

alpha and beta in the paper. The discussion of these two parameters in the paper is based on a fixed learning rate.

4. In this paper, standard data sets are used to ensure that the comparative experiments are on the same track.

5. At the end of the introduction, some descriptions of the effects of the model are added.

6. We sincerely thank the reviewer for careful reading. As suggested by the reviewer, We have unified several cases in the paper, such as "GraphRel" and "CMAN"; Correction of punctuation errors; Modified "precision" instead of "accuracy"; Fixed rendering issues; Several wording changes were made using more common statements.

Thank you again for your positive comments on our manuscript. PLOS ONE is an influential magazine. I hope our articles can be published in your magazine. If there are any other corrections, please feel free to contact us.
