# Supplementary material for: Span-based single-stage joint entity-relation extraction model
Source: PLoS One. 2023 Feb 7;18(2):e0281055. doi: 10.1371/journal.pone.0281055 (PMC9904475; doi:10.1371/journal.pone.0281055)
Supplement: Attachment — Submitted filename: Response to Reviewers.pdf [file pone.0281055.s003.pdf]

Editor-in-Chief of *PLOS ONE*

Dear editor and reviewers

On behalf of all the contributing authors, I would like to express our sincere appreciations of your letter and reviewers' constructive comments concerning our article entitled "Span-Based Single-Stage Joint Entity-Relation Extraction Model" (Manuscript No.: PONE-D-22-22843R1). These comments are all valuable and helpful for improving our article. According to the associate editor and reviewers' comments, we have made extensive modifications to our manuscript and supplemented extra data to make our results convincing. In this revised version, changes to our "Revised Manuscript with Track Changes" were all highlighted within the document by using yellow-colored text. Point-by-point responses to the nice associate editor and two nice reviewers are listed below this letter.

To Reviewer #2:

A. We are sorry that we failed to complete the statistical significance test before. In order to prove that the model results do not come from random fluctuations, we have added ablation experiments in this revision to ensure the effectiveness of each component of the model.
B. Tab 6 has been revised and redundant experiments have been removed to make it clear.

Thank you again for your positive comments on our manuscript. PLOS ONE is an influential magazine. I hope our articles can be published in your magazine. If there are any other corrections, please feel free to contact us.
